# Molecular mechanisms of *Piriformospora indica* mediated growth promotion in plants

**DOI:** 10.1080/15592324.2022.2096785

**Published:** 2022-07-09

**Authors:** Anish Kundu, Jyothilakshmi Vadassery

**Affiliations:** Chemical Ecology Laboratory, National Institute of Plant Genome Research (NIPGR), New Delhi, India

**Keywords:** *Piriformospora indica*, plant–fungal interaction, plant growth, endophytes

## Abstract

*Piriformospora indica* is a root endophyte having a vast host range in plants. Plant growth promotion is a hallmark of the symbiotic interaction of *P. indica* with its hosts. As a plant growth-promoting microorganism, it is important to know the mechanisms involved in growth induction. Hitherto, multiple reports have demonstrated various molecular mechanisms of *P. indica*-mediated growth promotion, including protein kinase-mediated pathway, enhanced nutrient uptake and polyamine-mediated growth phytohormone elevation. Here, we briefly present a discussion on the state-of-the-art molecular mechanisms of *P. indica*-mediated growth promotion in host plants, in order to obtain a future prospect on utilization of this microorganism for sustainable agriculture.

## Introduction

*Piriformospora indica* (syn. *Serendipita indica*, Basidiomycota), a root endophytic fungus, exhibits an extensive host range including monocots and dicots.^1–3^ *P. indica* colonization is observed mostly in the root epidermis and cortex of most of the host plants, including Arabidopsis, maize, tobacco, barley, rice and poplar.^1,4–6^ In most of the hosts, it significantly stimulates growth, including biomass and seed production.^7–10^ Apart from growth promotion, this endophyte increases defense against microbial pathogens by altering microbial defense signaling pathways as well as defense phytohormones levels.^11,12^ Moreover, *P. indica* helps plants in tolerance of abiotic stresses, such as salinity, water stress, drought, low temperature and heavy metal toxicity.^13–18^ Hitherto, the overall impression of *P. indica* colonization signifies beneficial interactions with the hosts, indicating involvement of general recognition and signaling pathways.^9^ Furthermore, an elevation of the intracellular calcium concentration in the root cells with this fungus and fungal exudate indicated a trigger of intracellular signaling cascade upon plant–fungal interaction.^18,19,20^ However, very little is understood about the mechanisms of growth promotion in plants upon *P. indica* colonization; here, we have concisely compiled and focused on recent major explorations of molecular mechanisms of *P. indica*-mediated growth induction in plants.

### OXI1 kinase pathway mediates Piriformospora indica-induced growth promotion

OXI1 (OXIDATIVE SIGNAL INDUCIBLE1), an oxidative stress responsive serine/threonine protein kinase gene (At3g25250) in plant, plays an important role in pathogen response and is regulated by H_2_O_2_ and PDK1 (3-PHOSPHOINOSITIDE-DEPENDENT PROTEIN KINASE1), another serine/threonine kinase in eukaryotes involved in auxin transports and vascular development in plants.^19,21,22^

**Figure 1.**
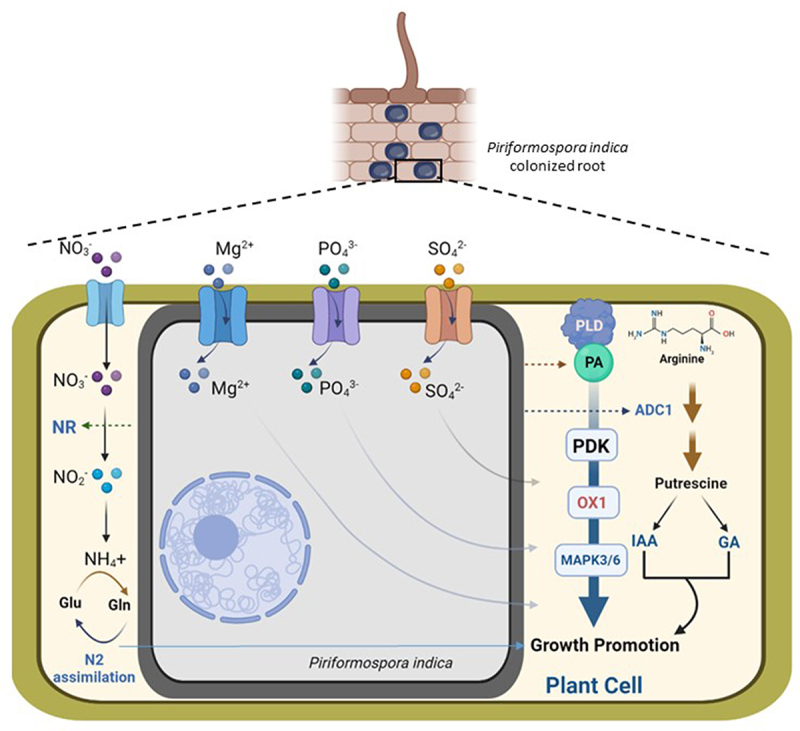
Schematic representation of cumulative molecular mechanisms of *P. indica*-mediated growth promotion in plants. Nitrates (NO_3_^−^) are transported in the *P. indica*-colonized plant cell and are converted to nitrites (NO_2_^−^), which are further used in nitrogen (N_2_) assimilation. *P. indica* induces nitrate reductase (NR) activity that enhances N_2_ assimilation. Nutrient transporters (Mg^2+^, PO_4_^3-^ and SO_4_^2-^ transporters) induce nutrients uptake in *P. indica*-colonized root and promotes growth in plants. But how these nutrients are transported to the host cell is not known; therefore, simple arrows are used to indicate their transportations from the *P. indica* cell to the plant host cell. *P. indica* mediates the induction of phospholipase D-mediated biosynthesis of phosphatidic acid (PA), which activates cascade of PDK1 (3-PHOSPHOINOSITIDE-DEPENDENT PROTEIN KINASE1), OXI1 (OXIDATIVE SIGNAL INDUCIBLE1) and MAPK3/6 (MAP Kinase 3/6) and results in growth promotion of plants. *P. indica* induces ADC1 (ARGININE DECARBOXYLASE 1)-mediated biosynthesis of putrescine, which elevates IAA (indole-3-acetic acid) and GAs (gibberellins) in host plant, which promotes growth.

In search of genes responsible for *P. indica*-induced growth response, Camehl et al., 2011 genetically screened Arabidopsis, which revealed *P. indica* to show impaired growth promotion in OXI1 and PDK1 loss-of-function mutants,^9^ and both *OXI1* (and an homologue *AGC2-2*) and *PDK1* transcript levels were upregulated upon *P. indica* colonization in Arabidopsis. They also demonstrated that a phospholipase D (PLD) facilitated a cascade consisting PDK1 and OXI1 (and AGC2-2) where *P. indica* induces PLD-mediated biosynthesis of phosphatidic acid (PA) and PA activates PDK1, which results in the induction of OXI1 and AGC2-2 triggering MAPK 3/6 (MAP kinases) (Figure 1). This whole cascade is required for beneficial interaction, especially *P. indica*-mediated growth promotion in Arabidopsis.

### *P*. *indica improves some of the macronutrients transport inside the plant cell and induces growth promotion*

A foremost cause for *P. indica*-induced plant growth promotion is increased nutrient uptake in the host plants.^23–29^ During colonization in roots, *P. indica* elevates nutrient uptake in host plants even in a low nutrient condition. The fungi contain a high-affinity phosphate (PO_4_^3-^) transporter (PiPT), which belongs to the phosphate: H1 symporter (PHS) family within the Major Facilitator Superfamily (MFS) and the crystal structure of this transporter with inorganic phosphate were solved by Pederson et al., 2013.^23^ They demonstrated a 2.9A° structure of a fungal transporter in an inward-facing occluded state where the phosphate was visible in the membrane-buried binding site. This *P. indica* transporter improves the phosphate intake of plants (Figure 1), but *P. indica* does not induce plant’s own phosphate uptake capabilities.^24^

Nitrogen is one of the key macronutrients for plant’s growth and development, which is utilized by plants majorly in the form of nitrate (NO_3_^−^) or ammonium (NH_4_^+^). Sometimes, limited nitrogen source may affect the plant’s growth. In a report, it was demonstrated that *P. indica* stimulates nitrogen accumulation in the roots of Arabidopsis and tobacco seedlings by inducing nitrate reductase and the starch-degrading enzyme, glucan-water dikinase (SEX1), resulting in growth promotion.^25^ *P. indica* was also found to improve nitrogen acquisition during colonization in tomatoes, which helps in host’s growth and development.^26^ Nitrogen was transported from the culture medium to *P.indica*, which was further transferred to the host. It was also hypothesized that hosts control the amount of nitrogen transferred from the fungi.^26^ Nevertheless, further studies are needed to explore the molecular and physiological mechanism of transportation regulation.

Subsequently, *P. indica* was also found to increase magnesium (Mg^2+^), an important macronutrient, uptake in Arabidopsis, which results in growth promotion of the plants (Figure 1). In search of the mechanism involved in increased Mg^2+^ uptake, a magnesium transporter named *Pi*MgT1 was determined and functionally characterized in *P. indica*, which belongs to the CorA-like protein family of bacteria.^27^ *Pi*MgT1 showed complementation in a yeast magnesium transporter-mutant CM66, which confirmed its functionality by restoring the growth of Mg^2+^ transport mutants, lacking the plasma membrane-localized Mg^2+^ transporter genes, ALR1 and ALR2.

In a recent report, a sulfur-specific high-affinity transporter, *Si*SulT, has been identified and characterized in *P. indica* during interaction with maize.^28^ This transporter has been demonstrated to be crucial for sulfur uptake in the sulfate form (SO_4_^2-^) to the host plant and promoting growth and development of the host under low-sulfate conditions (Figure 1). During transport of the sulfur, *P. indica* also induces the expression of host’s genes related to the sulfur assimilation pathways, indicating alteration of overall sulfur metabolism in a beneficial way to the host plant upon colonization.^29^

Carbohydrate or sugar is one of the most important primary metabolite groups for growth and development in plants. Genome sequencing of *P. indica* identified 19 putative hexose transporter genes, which show 15–91% homology with each other;^30^ among them, a *Pi*HXT5 hexose transporter was characterized during interaction with maize (Figure 1). This fungal transporter was 64-fold upregulated during colonization inside the host as compared to the axenic state, which indicates that symbiotic signaling controls the expression of this transporter. Its expression at a low glucose concentration revealed its high-affinity nature and host’s cellular glucose concentration dependency. This transporter belongs to the MFS superfamily with 12 predicted transmembrane helices. However, the relationship between host’s photosynthetic activity and the transcript level of fungal transporter and the correlation of symbiotic signaling and glucose signaling have not been revealed yet.^30^ It is also not known whether *P. indica* with its accumulated sugars can help the host in the extreme sugar-deficient environment. Therefore, more research is required to comprehend *P. indica*’s role in sugar transportation in the host plants and whether it truly takes part in the growth promotion.

Among the macronutrients required for uninterrupted plant growth, potassium (K^+^) is crucial. A study on *P. indica*-mediated alteration of K^+^ transportation in Arabidopsis exhibited the presence of multiple K^+^ transporters in the *P. indica*, i.e. *SiHAK1, SiTRK1, SiTRK2* and *SiTOK1*.^31^ Nonetheless, in the low K^+^ condition *P. indica* did not promote the growth of Arabidopsis. Surprisingly, under the condition of K^+^ deficiency, *P. indica* reduces overall K^+^ accumulation in the host plant while improving its own growth by expressing its own K^+^ transporter gene *SiHAK1*. Therefore, *P. indica* does not improve K^+^ transportation or accumulation inside the host cell for growth promotion.^30^ Furthermore, it was demonstrated that, under salt stress, *P. indica* induces K^+^ concentration in the host, which does not take part in growth promotion but alters Na^+^/ K^+^ homeostasis.^32^

### P. indica induces putrescine-mediated elevation of growth phytohormones that promotes plant growth

*P. indica* alters multiple phytohormonal regulated pathways during colonization, e.g. jasmonate-regulated secondary metabolite, glucosinolate pathway during early stages of interaction,^31^ rapid increase in auxin levels during early recognition of the host, which is important for reprogramming the root development,^33^ - elevated level of auxins in Arabidopsis roots,^5^ and *P. indica* itself also produces IAA in the liquid culture.^34^ In a recent study, it has been elucidated that *P. indica* employs putrescine for plant’s growth promotion.^10^ *P. indica* induces putrescine biosynthesis in tomato (*Solanum lycopersicum*) through the ARGININE DECARBOXYLASE 1 (*Sl*ADC1)-mediated pathway, as metabolomics and gene expression data indicated that *P. indica* induces putrescine content and *SlADC1* transcript level in tomato upon colonization. Growth promotion with *P. indica* was found to be impaired in *SlADC1*-suppressed or -mutant lines of tomato and/or Arabidopsis, and the growth promotion was restored by complementation with exogenous putrescine supplementation. Exogenous putrescine treatment also elevates auxin (indole-3-acetic acid) and specific gibberellins (GA_4_ and GA_7_) in tomato, which suggests that *P. indica* induces putrescine to elevate growth phytohormone levels, which finally results in plant’s growth promotion^10^ (Figure 1).

## Conclusion

*P. indica*-mediated growth promotion in plants is a crucial area in plant biology as it will have a great impact on agriculture to improve crop yield in the future. However, a few major physiological mechanisms of growth induction have been explored, and the complexity and characteristics of this mutualistic interaction of *P. indica* vary greatly from host to host. Therefore, there are possibilities to have host-specific mechanisms of *P. indica*-mediated growth induction, which has to be explored. There are major gaps in the information regarding *P. indica*-mediated nutrient transportation. For example, although *P. indica* induces the nutrient uptake through nutrient transporters, the mechanism of nutrient transportation to the plant cell from fungal spores or hyphae is not clear yet, which has to be elucidated. Hexose transporter characterized in the *P. indica* helps the fungi to accumulate sugars in the low sugar condition of the host; however, it is not revealed that whether these accumulated sugars are further transferred to the host’s cell for aiding it to overcome the sugar deficiency. Apart from this, multiple questions have been raised from the current information regarding *P. indica*-mediated growth promotion, such as how the expressions of nutrient transporters are being regulated upon establishment of *P. indica* colonization in host?, whether *P. indica* only induces sugar uptake or does it also induce sugar biosynthesis in the host? How does putrescine induce growth phytohormone levels in the *P. indica*-colonized plants? All these questions need to be answered to shed more light on this complex mechanism of plant’s growth promotion.
